# Chronic Isolation Stress Affects Subsequent Crowding Stress-Induced Brain Nitric Oxide Synthase (NOS) Isoforms and Hypothalamic-Pituitary-Adrenal (HPA) Axis Responses

**DOI:** 10.1007/s12640-019-00067-1

**Published:** 2019-06-18

**Authors:** Anna Gądek-Michalska, Joanna Tadeusz, Andrzej Bugajski, Jan Bugajski

**Affiliations:** 1 Department of Physiology, Institute of Pharmacology, Polish Academy of Sciences, Smętna 12 Street, 31-343 Kraków, Poland; 2 Department of Pathophysiology, Jagiellonian University Medical College, Czysta 18 Street, 31-121 Kraków, Poland

**Keywords:** Isolation and crowding stress, iNOS, nNOS, IL-1β, ACTH, Corticosterone

## Abstract

The nitric oxide (NO) pathway in the brain is involved in response to psychosocial stressors. The aim of this study was to elucidate the role of nNOS and iNOS in the prefrontal cortex (PFC), hippocampus (HIP), and hypothalamus (HYPO) during social isolation stress (IS), social crowding stress (CS), and a combined IS + CS. In the PFC, 3 days of CS increased iNOS but not nNOS protein level. In the HIP and HYPO, the levels of nNOS and iNOS significantly increased after 3 days of CS. In the PFC, IS alone (11 days) enhanced iNOS protein level following 3 days of CS and increased nNOS level in the HIP and HYPO after 14 days of CS. By contrast, in the HIP, IS abolished the subsequent CS-induced increase in nNOS in the HIP and strongly elevated iNOS level after 7 days of CS. In the HYPO, prior IS inhibited nNOS protein level induced by subsequent CS for 3 days, but increased nNOS protein level after longer exposure times to CS. Isolation stress strongly upregulated plasma interleukin-1β (IL-1β) and adrenocorticotropic hormone (ACTH) levels while corticosterone (CORT) level declined. We show that the modulatory action of the NO pathway and ACTH/CORT adaptation to chronic social isolation stress is dependent on the brain structure and nature and duration of the stressor. Our results indicate that isolation is a robust natural stressor in social animals; it enhances the NO pathway in the PFC and abolishes subsequent social CS-induced NOS responses in the HIP and HYPO.

## Introduction

The activation of the hypothalamic-pituitary-adrenal (HPA) axis is widely accepted as one of the central physiological mechanisms involved in the stress response. Chronic stressors in nature can activate direct physiological challenges and threaten homeostasis and do not require cognitive processing, while anticipatory stressors, perceived to be threatening, require cognitive processing (McEwen et al. 2015; Herman et al. 2003; Boonstra 2013). Chronic emotional, psychological stress represents one of the major environmental factors associated with adverse impact on mental health, mood disorders, and the development of comorbid psychiatric illnesses (Duric et al. 2016; Liu et al. 2013; Horowitz and Zunszain 2015).

The prefrontal cortex (PFC), the most highly evolved brain region, is also highly sensitive to the detrimental effects of stress (Arnsten 2009). The most adapting changes induced by chronic stress are observed in the hippocampus (HIP) and PFC—brain regions which play a role in mediating the effects of stress on the regulation of glucocorticoids (GCs) in rats similar to those observed in people with psychiatric disorders, including depression (Filipović et al. 2017).

Exposure to stress increases the production of nitric oxide (NO) in the brain. Nitric oxide synthases catalyze the production of NO from L-arginine; nitric oxide synthase (NOS) signaling pathway plays different roles during acute or chronic stress. Within biological systems, the concentration of NO is regulated by the activity of NOS isoforms: constitutively expressed neural (nNOS) and endothelial (eNOS) and inducible (iNOS) forms. Nitric oxide synthase-positive neurons are located in the HIP and cerebral cortex. Neuronal NOS activation by acute restraint stress plays a prominent role in local nitrergic neurotransmission in the hypothalamic paraventricular nucleus (PVN) while having a facilitatory influence on the delayed emotional consequences of stress (Busnardo et al. 2019). Nitric oxide generated by nNOS can act as a neurotransmitter (Guix et al. 2005). It is involved in the cholinergic stimulation of the HPA axis response during psychosocial crowding stress (CS) (Bugajski et al. 2006). Tropisetron, a serotonin 5-HT_3_ receptor antagonist, reversed the negative effects of iNOS on mitochondrial dysfunction in animal models of depression (Haj-Mirzaian et al. 2016a; Amiri et al. 2015). Inducible type NOS (iNOS), though rarely present in cells, is expressed in numerous cell types, mainly in microglia and astrocytes in the vicinity of synaptic elements and can release NO and affect synaptic transmission (Amitai 2010). NO is involved in maintaining synaptic plasticity in the neuronal structures of the CNS. Changes in synaptic strength determine information storage in the formation of long-term memory (Shefa et al. 2018). When produced in excess, NO may induce oxidative/nitrosative damage and is neurotoxic (Contestabile et al. 2012; Zlatković and Filipović 2013). Acute stressors are known to increase nNOS, but not iNOS protein expression in the PFC (Zlatković and Filipović 2013).

Stressors of the social nature in humans and social animals have a stronger damaging effect on health and mortality risk than other traditional risk factors (Moieni et al. 2015). Chronic social isolation is continuous and entirely different from the natural conditions of social animals living in groups (Filipović et al. 2017). Psychosocial relations determine the stress response in humans and social animals (Koolhaas et al. 1997; Bartolomucci et al. 2005; Cacioppo et al. 2015). Compared with other traditional stress models, negative social relationships may create marked chronic stress and neuro-hormonal dysregulations.

Group housing animals is helpful as it allows the animals to overcome stressful conditions, whereas individual housing is more stressful and enhances central and systemic responses of adaptation mechanisms eliciting the release of cytokines and the activation of the NO pathway. Neuronal and extraneuronal sources of NO synthesis are involved in social stress-induced responses. Chronic social isolation increases the expression of cytosolic nNOS and iNOS proteins in brain structures amenable to stress: PFC, HIP, and hypothalamus (HYPO) (Zlatković and Filipović 2013) through the activation of the NOS signaling pathway.

We found that chronic CS may protect against the possible harmful effects of hyperexpression of iNOS in the brain, particularly in the PFC and HIP. By contrast, repeated physico-psychosocial restraint stress may potentiate additional homotypic stress-induced effects of NOS and interleukin-1β (IL-1β) hyperproduction (Gądek-Michalska et al. 2016). It is well established that nNOS-mediated NO synthesis impairs hippocampal neurogenesis, which may be associated with the development of depression. Likewise, chronic mild stress (CMS) in mice induced prolonged overexpression of hippocampal nNOS and changes typical of depression. These changes were absent in mutant mice lacking the nNOS gene and in mice treated with a nNOS inhibitor (Chen et al. 2015; Spiers et al. 2016).

NO has a primarily excitatory role within the HPA axis and stimulates corticotropin-releasing hormone (CRH) secreting neurons in the PVN and median eminence (Busnardo et al. 2019; Bugajski 1999; Lee et al. 1999; Prevot et al. 2000; Coldren et al. 2017). Brain nNOS participates in the modulation of learning, memory, and neurogenesis, and is the predominant source of NO in the synaptic spines of neurons under basal and stress conditions. The mechanisms by which NO synthases modulate the stress response following CS and IS are largely unknown.

Increasing evidence suggests that the immune system is involved in the stress response and involve molecules such as CRH, ACTH, and NO which carry out both neuroendocrine and immune functions. A role for cytokines and NO in the stress response or immunity has been demonstrated in many species (Ottaviani and Franceschi 1996; Ottaviani et al. 2007).

A variety of experimental models using laboratory animals (mice, rats, guinea pigs, and dogs) have been evaluated for their response to stress (Garcia-Bueno et al. 2008). In the majority of these investigations evaluating the adaptation mechanisms of stress markers, prior chronic or repeated stress was followed by acute homotypic or heterotypic stressors. However, the effect of chronic psychosocial stress of isolation (strong stress) and subsequent crowding (mild stress) on the expression of NO synthases in the brain especially in stress-relevant structures like the PFC, HIP, and HYPO is unknown. The aim of the present study was to evaluate changes in nNOS and iNOS protein expression in these brain regions following the exposure of rats to chronic IS and CS of various durations. Two experimental models of psychosocial stress (CS and IS) were utilized to compare the effects of chronic IS on subsequent CS-induced nNOS and iNOS protein expression in the PFC, HIP, and HYPO. The combined action of the psychosocial stressors on the HPA axis stress mediators, plasma IL-1β, ACTH, and CORT levels was also investigated and compared with relevant changes of nNOS and iNOS in the brain structures.

## Materials and Methods

### Animals

All the experimental procedures were approved by the Committee for Laboratory Animal Welfare and the Ethics Committee of the Institute of Pharmacology, Polish Academy of Sciences in Kraków and were carried out in compliance with European Union legislation (Directive 2010/63/EU). Experiments were performed on unanesthetized male Wistar rats (6–7 weeks old, weighing 76–100 g at the beginning of the study) purchased from Charles River Laboratories, Sulzfeld, Germany. The animals were kept in standard cages (52 × 32 × 20 cm) and given unrestricted access to commercial rat chow and water. The animal room was maintained under standard laboratory conditions: an artificial 12/12 h light/dark cycle (lights on from 7 a.m. to 7 p.m.) and at constant room temperature (22 ± 2 °C). Before the experiments commenced, the animals were acclimatized to their new environment for 7 days.

### Experimental Procedures

The animals were randomly allocated to control groups or groups subjected to either crowding stress (CS), isolation stress (IS), or combined IS + CS. Animals in the control group (5–7 animals per cage) were not subjected to stress.

#### CS

Rats were randomly assigned to one of two experimental groups: control and social stress of crowding. Control rats were housed 7 per standard cage and remained in their home cages until scheduled for treatment. Stressed rats were crowded in groups of 24 per cage of the same size for 3, 7, or 14 consecutive days, enough time for potent impairment of the HPA axis response (Bugajski et al. 2002; Gądek-Michalska et al. 2017).

#### IS

Animals in the IS group were kept individually in standard cages for 11 days. A group of 7 animals per cage that were not subjected to stress served as controls. Different exposure periods (1–21 days) have been reported for social isolation (Zlatković et al. 2014) and 4 weeks for an animal model of depression (Pournajafi-Nazarloo et al. 2011). We chose an 11-day isolation stress period because it accommodates the recovery time after surgery and at the same exposes the animals living in groups to very severe psychosocial stress.

#### IS + CS

Animals were exposed to IS (one per cage for 11 days) and then immediately subjected to CS for 3, 7, or 14 days as described above.

### Blood and Tissue Collection

All animals were sacrificed by rapid decapitation (within few seconds after removal from the cage) on the last day of the experiment. Afterward, the brains were removed from the skulls and brain structures: PFC, HIP, and HYPO were dissected on an ice-cold glass plate, immediately frozen on dry ice and stored at − 70 °C until required for further studies. Trunk blood for plasma CORT and IL-1β determinations was collected in the presence of EDTA (10% *w*/*v*; Merck, Germany; 25 μl/ml of blood) and in the case of ACTH immunoassay, blood was drawn into tubes containing EDTA and aprotinin (0.6 TIU/ml of blood; Sigma-Aldrich, St. Louis, USA) and centrifuged at 1400 rpm for 10 min at 4 °C.

### Plasma Hormone and IL-1β Measurement

Plasma CORT, ACTH, and IL-1β concentrations were determined using the following kits: Rat/Mouse Corticosterone EIA kit (Immunodiagnostic Systems, Boldon, UK), ACTH Rat/Mouse EIA kit (Phoenix Pharmaceuticals, Burlingame, CA, USA) and Rat IL-1β ELISA kit (BioVendor, Brno, Czech Republic) following the manufacturer’s instructions. All the measurements were performed in duplicate.

### Protein Extract Preparation

Frozen brain tissue samples were homogenized in Eppendorf tubes (Ultra-Turrax, 10,000 rpm) containing an ice-cold solution of RIPA buffer (Sigma-Aldrich) and freshly added Protease Inhibitor Cocktail and Phosphatase Inhibitor Cocktail 2 and 3 (1:100, Sigma-Aldrich). Samples were then centrifuged at 14,000 rpm for 20 min at 4 °C. The protein concentrations of the samples were determined using the BCA™ Protein Assay Kit (Thermo Fisher Scientific, Waltham, MA, USA). Appropriate concentrations of the homogenates were mixed 1:1 with Laemmli sample buffer (Bio-Rad, Hercules, USA) and β-mercaptoethanol (50 μl per 950 ml of Laemmli buffer; Sigma-Aldrich) and boiled for 5 min.

#### SDS-PAGE Gel Electrophoresis and Western Blot

Homogenates were assayed for nNOS and iNOS protein levels by sodium dodecyl sulfate polyacrylamide gel electrophoresis and western blot procedures as previously described (Gądek-Michalska et al. 2015). Blots containing proteins were exposed to the following antibodies: primary rabbit anti-nNOS (1:400, sc-648) and anti-iNOS (1:400, sc-650), polyclonal antibodies, primary mouse anti-β-actin (1:400, sc-47778), monoclonal antibody followed by goat anti-rabbit (1:10000, sc-2004), and goat anti-mouse (1:4000, sc-648) horseradish peroxidase–conjugated secondary antibodies, all of which were provided by Santa Cruz Biotechnology, Dallas, TX, USA. Proteins were visualized by enhanced chemiluminescence (Lumi-LightPlus Western Blotting Kit, Roche Diagnostics, Switzerland). Immunoblots were subsequently evaluated using a luminescent image analyzer (LAS-4000, Fujifilm, Japan). The optical densities of the appropriate bands were quantified by densitometry using Image Gauge V4.0 software (Fujifilm, Japan) and normalized to β-actin levels. All the values are expressed as a percentage of controls.

#### Statistical Analysis

Results are presented as means ± standard error of the mean (S.E.M.) where *n* = 10–12 rats per group. Statistical analyses were conducted using GraphPad Prism 7.04 (GraphPad Software Inc., USA). Data analysis was performed by one-way analysis of variance (ANOVA) followed by Newman’s test. Groups that were significantly different from the non-stressed control group are indicated in the figures as follows: ^+^*p* < 0.05, ^++^*p* < 0.01, ^+++^*p* < 0.001, ** *p* < 0. 01, ***p < 0.001 vs. isolation stress group, and in the case of a two-way ANOVA, it was followed by a post hoc Tukey’s multiple comparison test: *p < 0.05, **p < 0.01, ***p < 0.001 vs. IS + CS/IS group; ^#^*p* < 0.05, ^##^*p* < 0.01, ^###^*p* < 0.001 vs. IS + CS/CS group. The data were also analyzed by Student’s *t* test (^++^*p* < 0.01 and ^+++^*p* < 0.001 vs. non-stressed control group).

## Results

### Effect of Social CS on nNOS and iNOS Protein Levels

**Fig. 1:**
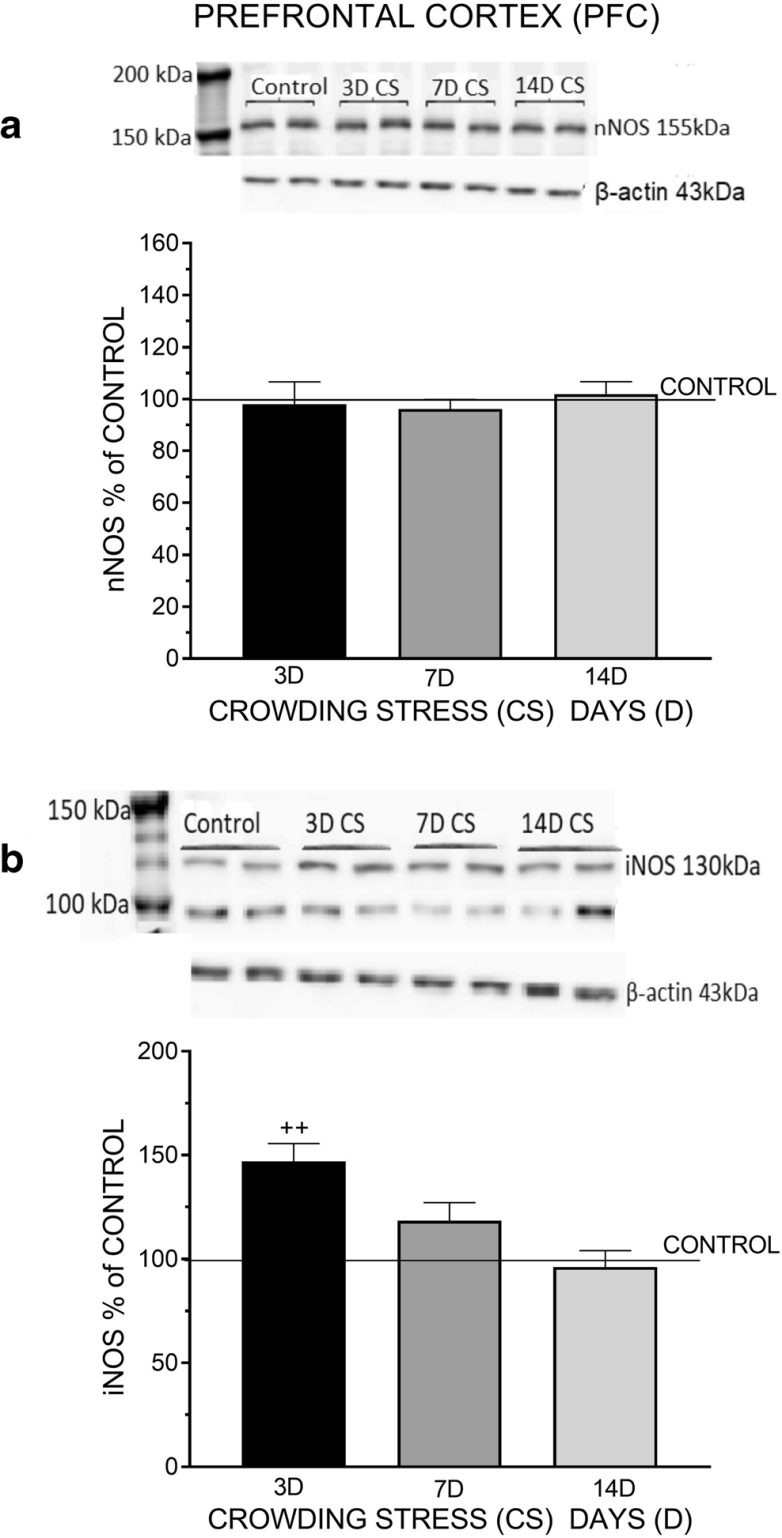
The effect of crowding stress (CS) for 3, 7, and 14 days on nNOS (**a**) and iNOS (**b**) levels in the prefrontal cortex. Rats were exposed to crowding stress (24 animals per cage) for 3, 7, and 14 consecutive days and decapitated. The panels above show representative immunoblots showing the expression of nNOS (**a**) and iNOS (**b**) in the prefrontal cortex. Graphs represent the means ± SEM of 10–12 rats per group. Data were assessed by one-way ANOVA followed by Newman’s test: ++ *p* < 0.01 vs. non-stressed control group

**Fig. 2:**
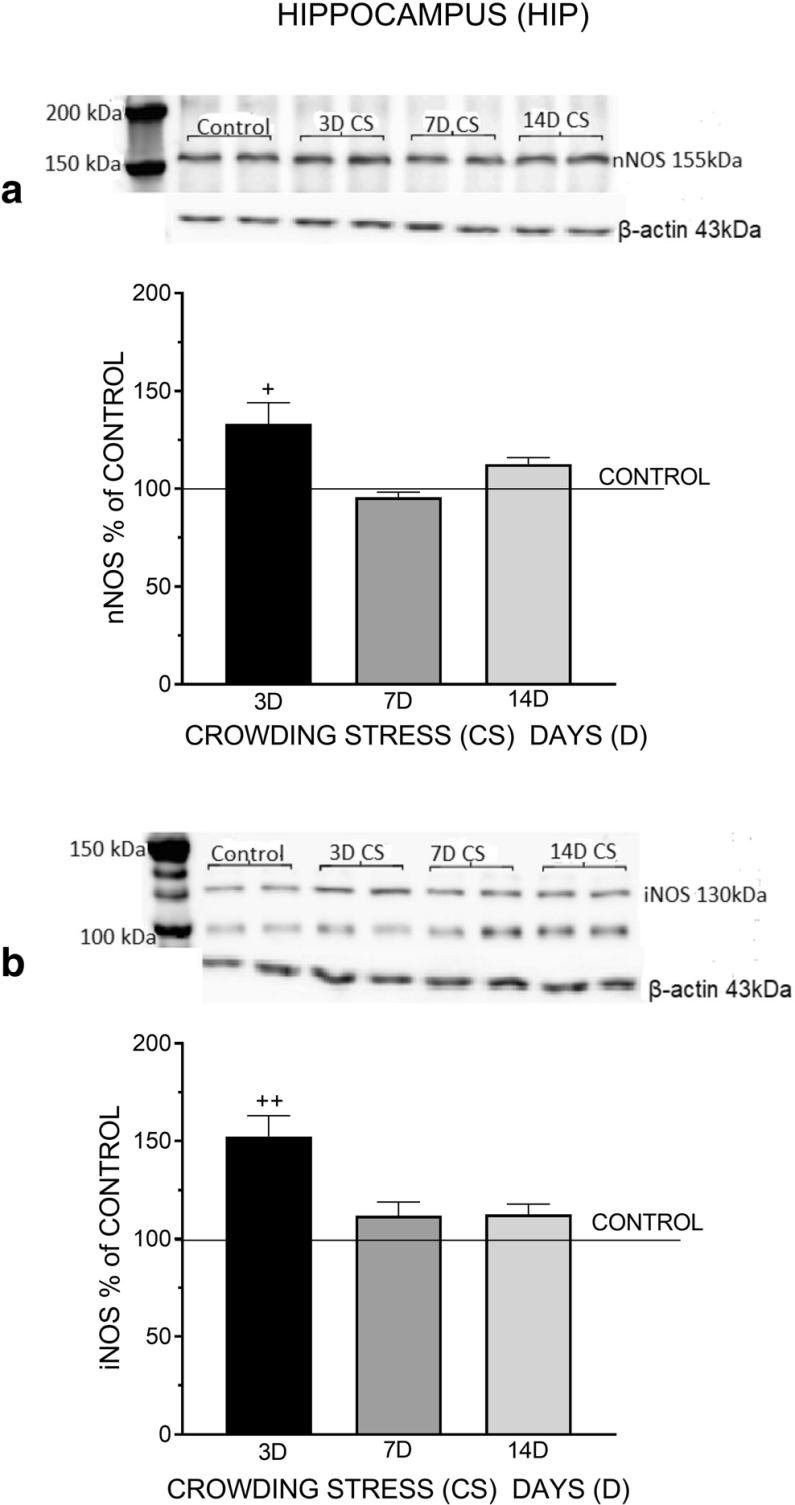
The effect of crowding stress (CS) for 3, 7, and 14 days on nNOS (**a**) and iNOS (**b**) levels in the hippocampus. Rats were exposed to crowding stress (24 animals per cage) for 3, 7, and 14 consecutive days and decapitated. The panels above show representative immunoblots showing the expression of nNOS (**a**) and iNOS (**b**) in the hippocampus. Graphs represent the means ± SEM of 10–12 rats per group. Data were assessed by one-way ANOVA followed by Newman’s test: ^+^*p* < 0.05 and ^++^*p* < 0.01 vs. non-stressed control group

**Fig. 3:**
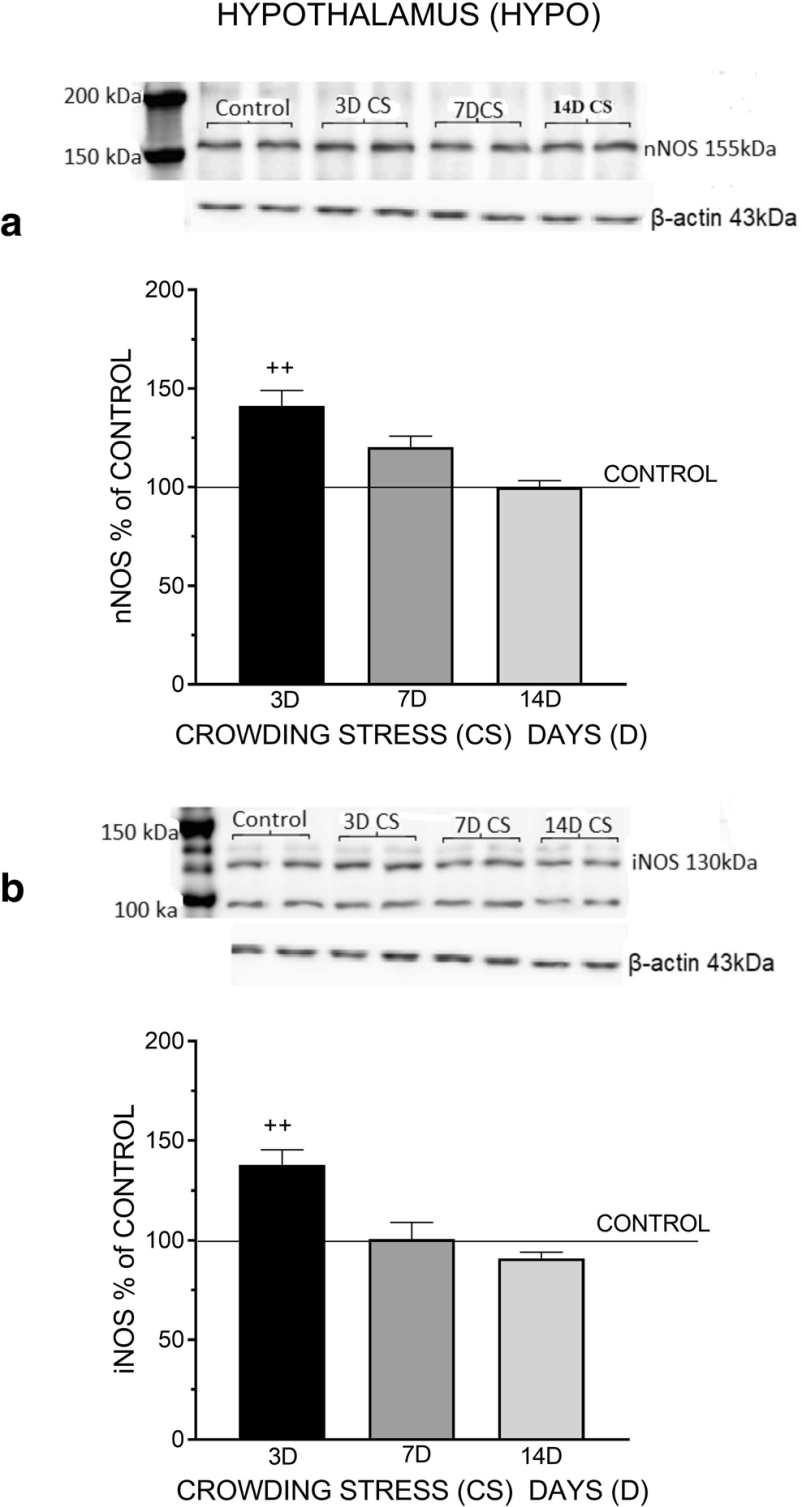
The effect of crowding stress (CS) for 3, 7, and 14 days on nNOS (**a**) and iNOS (**b**) levels in the hypothalamus. Rats were exposed to crowding stress (24 animals per cage) for 3, 7, and 14 consecutive days and decapitated. The panels above show representative immunoblots showing the expression of nNOS (**a**) and iNOS (**b**) in the hypothalamus. Graphs represent the means ± SEM of 10–12 rats per group. Data were assessed by one-way ANOVA followed by Newman’s test: ++ *p* < 0.01 vs. non-stressed control group

In the PFC, social CS for 3, 7, and 14 days did not alter nNOS protein level markedly compared with the resting level in non-stressed rats (*F*_(3,42)_ = 0.5784, *p* = 0.6324) (Fig. 1a). By contrast, CS for 3 days significantly increased iNOS protein level in the PFC (*F*_(3,33)_ = 7.711, ^++^*p* < 0.01) (Fig. 1b). In the HIP, CS for 3 days significantly increased nNOS protein level (*F*_(3, 52)_ = 4.588, ^+^*p* < 0.05); surprisingly, protein levels were not substantially altered after 7 and 14 days (Fig. 2a). Likewise, CS for 3 days strongly enhanced the expression of iNOS protein (*F*_(3, 44)_ = 5.725, ^++^*p* < 0.01); after 7 and 14 days iNOS levels were slightly elevated (Fig. 2b). In the HYPO, CS for 3 days significantly increased nNOS (*F*_(3, 42)_ = 6.756, ^++^*p* < 0.01) and iNOS protein levels (*F*_(3, 42)_ = 5.18, ^++^*p* < 0.01), but did not alter the levels of these proteins markedly after 7- and 14-day exposure to CS (Fig. 3a, b).

### Effect of Chronic IS on nNOS and iNOS Protein Levels

**Fig. 4:**
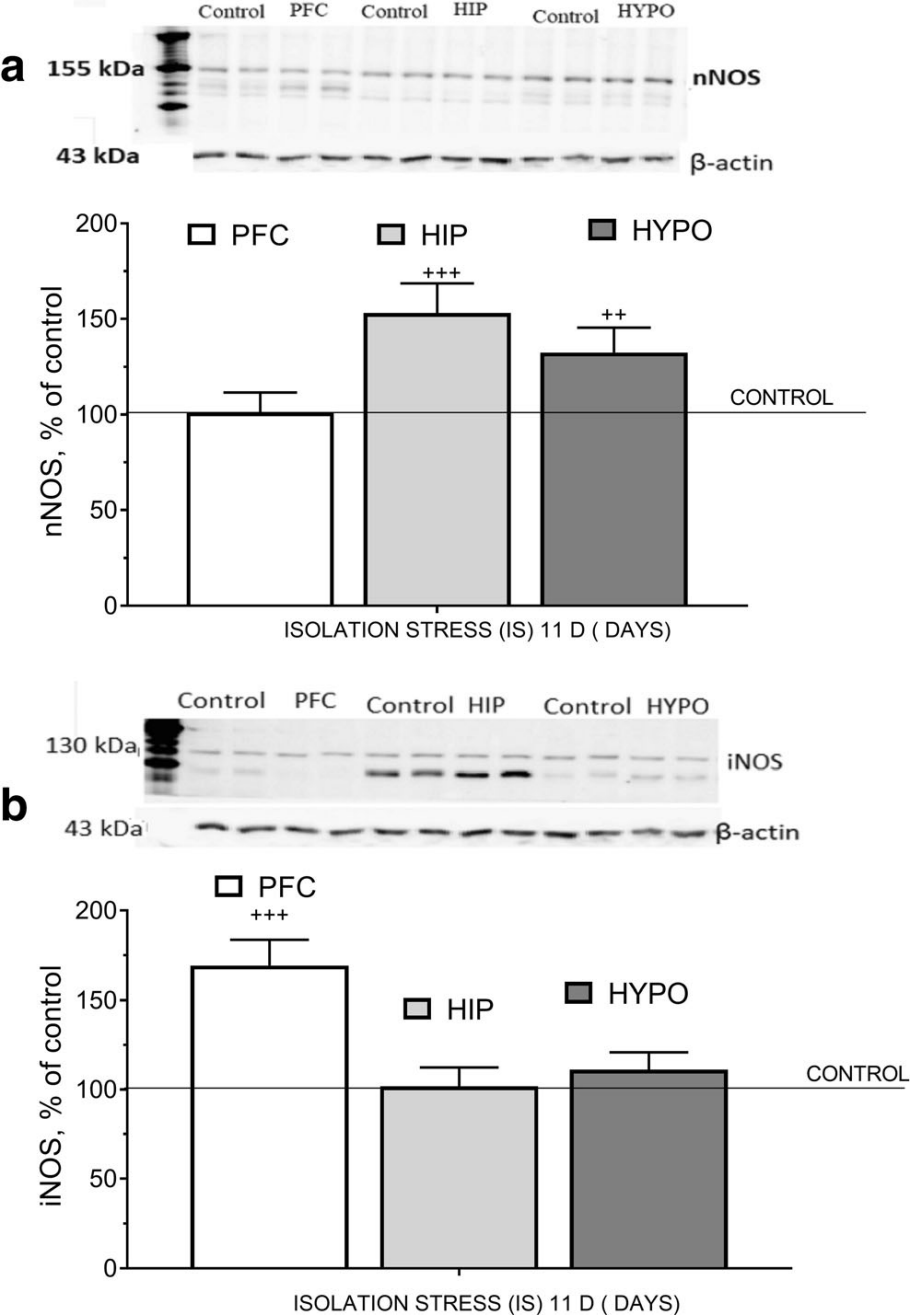
The effect of isolation stress (IS) for 11 days on nNOS (**a**) and iNOS (**b**) levels in the prefrontal cortex (PFC), hippocampus (HIP), and hypothalamus (HYPO). Rats were exposed to isolation stress (rats were kept 1 per cage for 11 consecutive days) and decapitated. The panels above show representative immunoblots showing the expression of nNOS (**a**) and iNOS (**b**) in the prefrontal cortex, hippocampus, and hypothalamus. Graphs represent the mean ± SEM, *n* = 10–12 rats per group. Data were assessed by Student’s *t* test: ^++^*p* < 0.01 and ^+++^*p* < 0.001 vs. non-stressed control group

In the PFC, IS for 11 days did not change nNOS protein level (*t* = 0.4052, df = 37, *p* = 0.6877) but significantly increased iNOS protein level (*t* = 5.463, df = 29, ^+++^*p* < 0.001) (Fig. 4a, b). In the HIP, 11 days IS significantly increased the expression of nNOS protein (*t* = 4.573, df = 34, ^+++^*p* < 0.001) but did not alter iNOS protein level (*t* = 0.2759, df = 35, *p* = 0.7842) (Fig. 4a, b). In the HYPO, IS for 11 days significantly elevated nNOS protein level (*t* = 4.106, df = 34, ^++^*p* < 0.01) but did not alter iNOS level (*t* = 1.145, df = 26, *p* = 0.2628) (Fig. 4a, b).

### Effect of Chronic IS on Subsequent CS-Induced nNOS and iNOS Levels

**Fig. 5:**
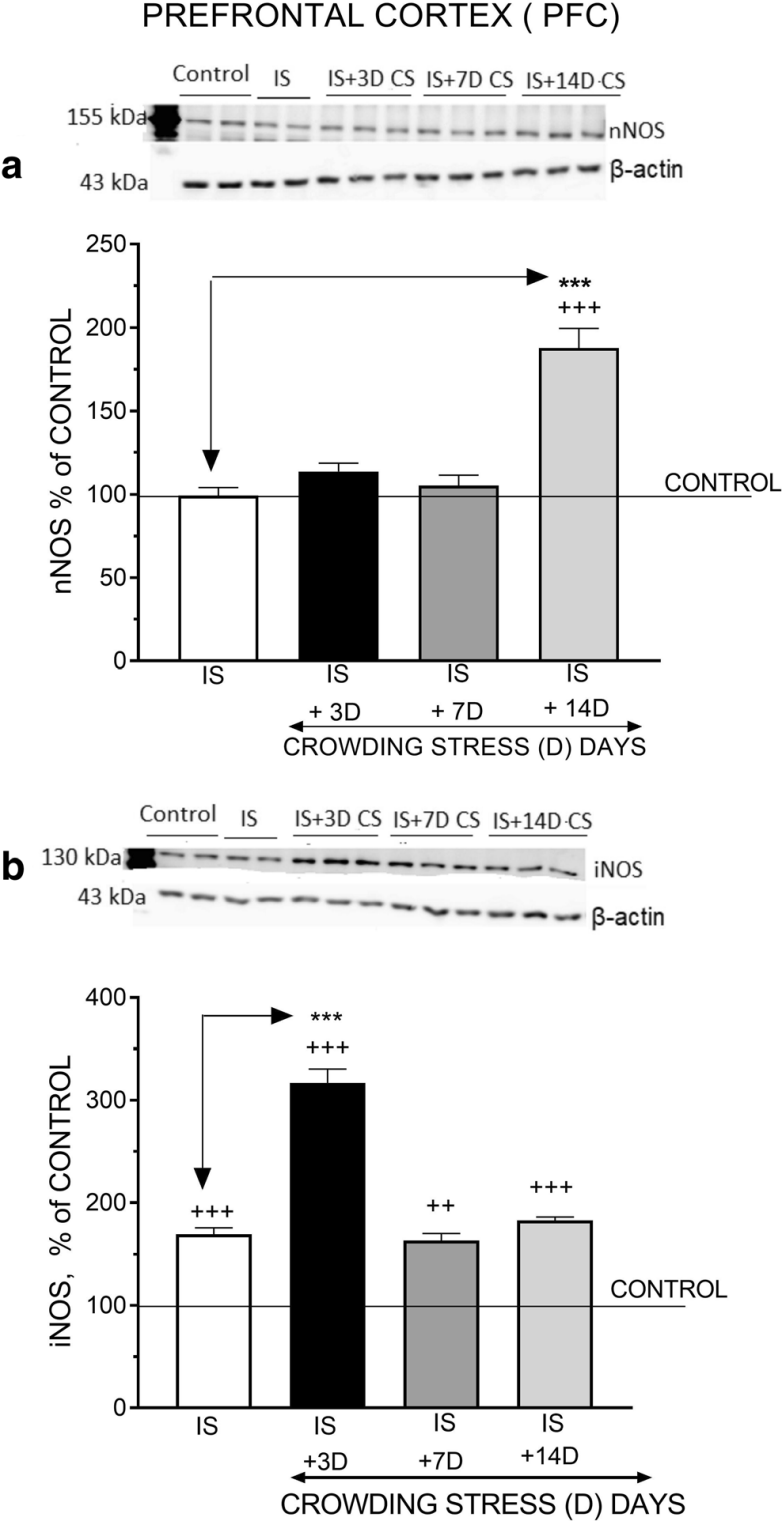
The effect of isolation stress (IS) for 11 days on subsequent crowding stress (CS) for 3, 7, and 14 days on nNOS (**a**) and iNOS (**b**) levels in the prefrontal cortex. Rats were exposed to isolation stress followed by crowding stress and decapitated. The panels above show representative immunoblots showing the expression of nNOS (**a**) and iNOS (**b**) in the prefrontal cortex. Graphs represent the means ± SEM of 10–12 rats per group. Data were assessed by one-way ANOVA followed by Newman’s test: ^++^*p* < 0.01 and ^+++^*p* < 0.001 vs. non-stressed control group; ***p < 0.001 vs. isolation stress

Rats initially exposed to 11 days IS and subsequently to CS for 3 and 7 days did not exhibit any alterations in nNOS protein level in the PFC. However, after exposure to CS for 14 days, nNOS protein level increased considerably in the PFC compared with the levels in non-stressed (*F*_(4.56)_ = 10.01, +++*p* < 0.001) and IS only treated controls (*F*_(3,46)_ = 11.93, ***p < 0.001) (Fig. 5a). IS alone for 11 days significantly increased iNOS protein level in the PFC compared with the level in non-stressed controls (*F*_(4,51)_ = 26.85, ^+++^*p* < 0.001) (Fig. 5b). Eleven days of IS followed by CS for 3 days considerably enhanced the increase of iNOS protein (*F*_(3,54)_ = 23.55, ***p < 0.001) compared with the non-stressed group, but did not alter iNOS protein levels after 7 and 14 days of subsequent CS compared with the levels after IS alone (Fig. 5b).

**Fig. 6:**
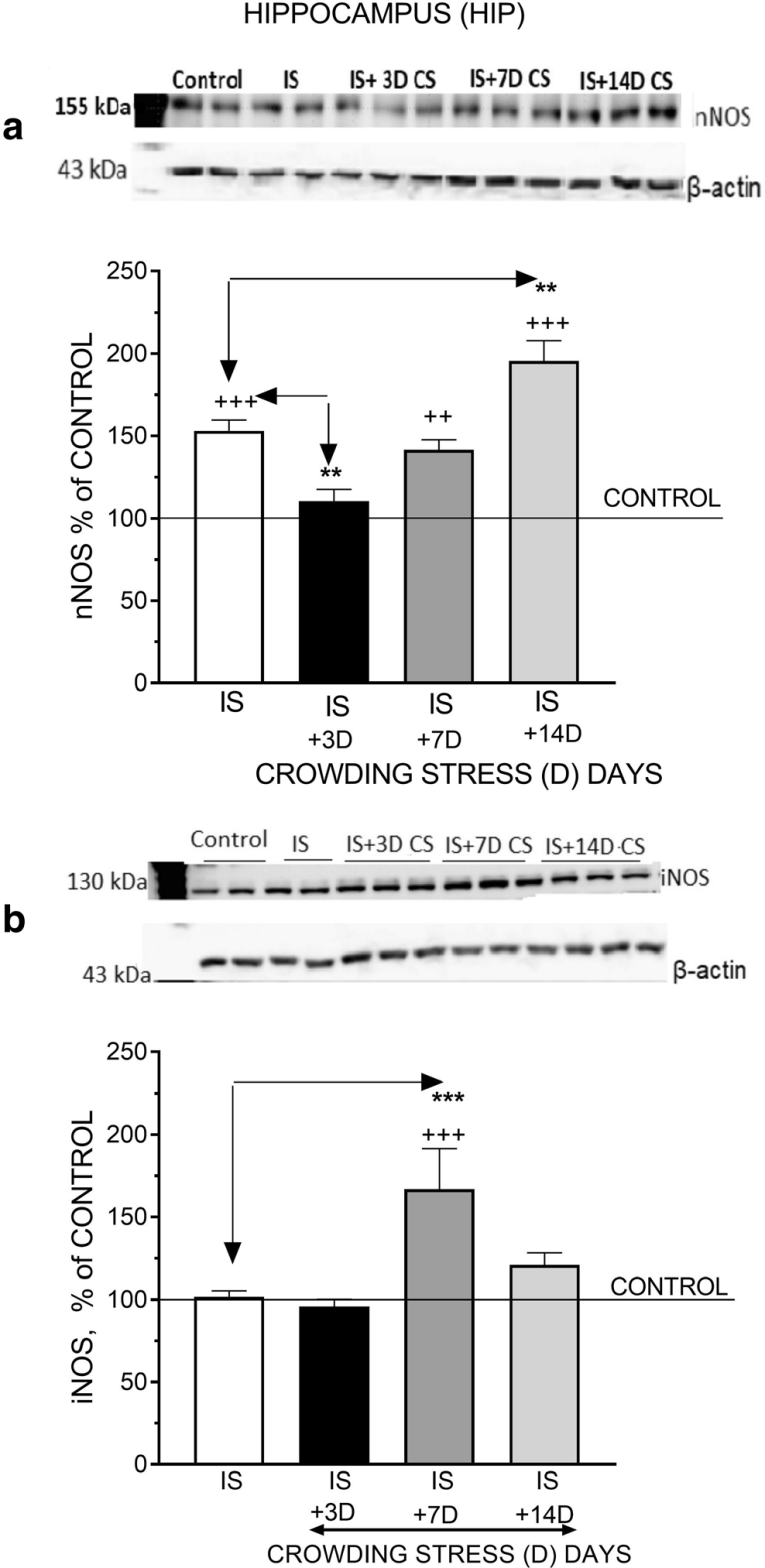
The effect of isolation stress (IS) for 11 days on subsequent crowding stress (CS) for 3, 7, and 14 days on nNOS (**a**) and iNOS (**b**) levels in the hippocampus. Animals were exposed to isolation stress followed by crowding stress and decapitated. The panels above show representative immunoblots showing the expression of nNOS (**a**) and iNOS (**b**) in the hippocampus. Graphs represent the means ± SEM of 10–12 rats per group. Data were assessed by one-way ANOVA followed by Newman’s test: ^++^*p* < 0.01 and ^+++^*p* < 0.001 vs. non-stressed control group; **p < 0.01 and ***p < 0.001 vs. isolation stress

In the HIP, IS alone for 11 days significantly increased nNOS protein level vs. control level in non-stressed rats (*F*_(4, 51)_ = 17.74, ^+++^*p* < 0.001), subsequent CS for 3 days reduced nNOS protein level significantly (*F*_(3, 31)_ = 12.06, **p < 0.01)*.* CS for 7 days did not alter nNOS protein level induced by IS markedly but CS for 14 days significantly enhanced nNOS protein level compared with the level induced by IS alone **p < 0.01 (Fig. 6a).

In the HIP, IS alone did not alter control iNOS protein level; however, subsequent CS for 7 days significantly increased iNOS level compared with control (*F*_(4,51)_ = 6.612, ^+++^*p* < 0.001), and IS alone (*F*_(3,49)_ = 8.135, ***p < 0.001). IS followed by 3 and 14 days of subsequent CS did not alter iNOS levels (Fig. 6b).

**Fig. 7:**
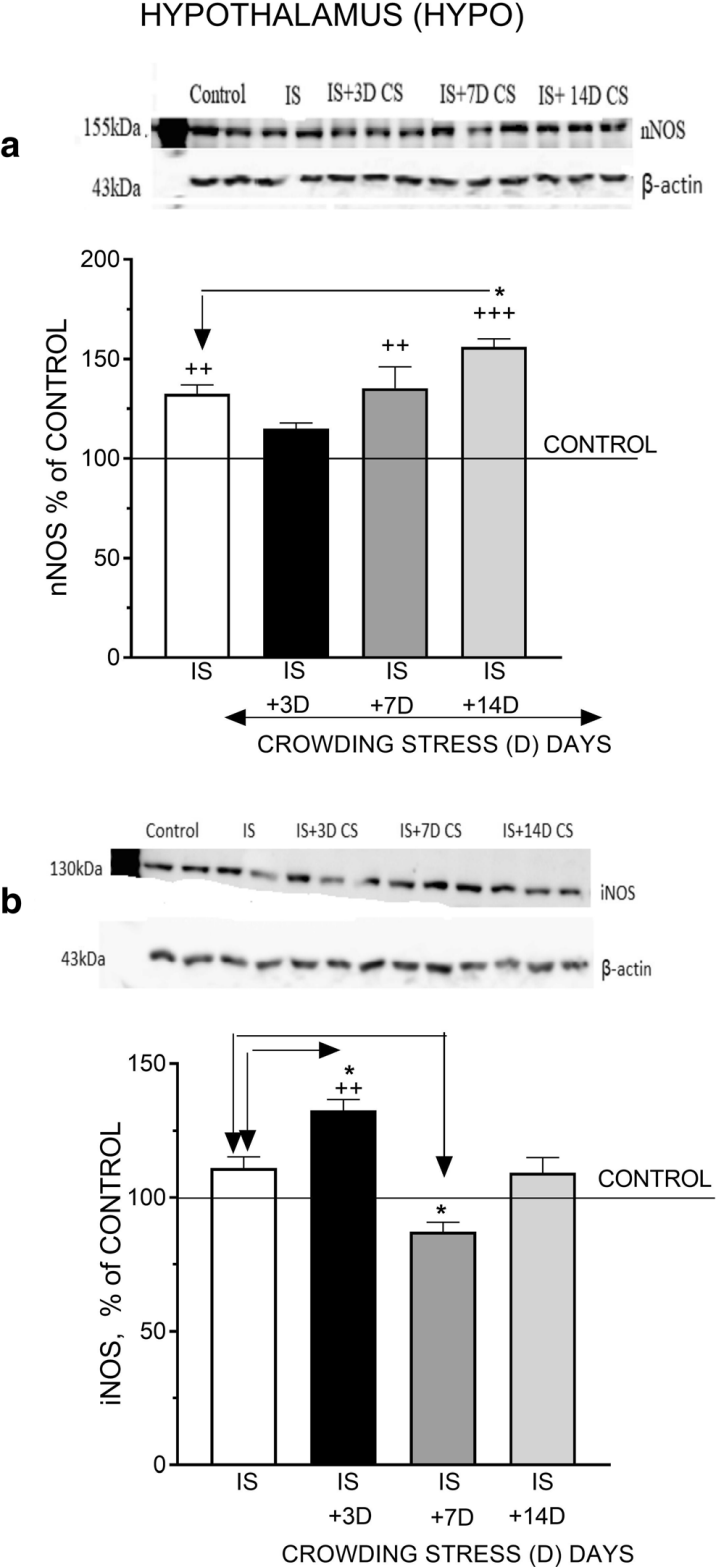
The effect of isolation stress (IS) for 11 days on subsequent crowding stress (CS) for 3, 7, and 14 days on nNOS (**a**) and iNOS (**b**) levels in the hypothalamus. Animals were exposed to isolation stress followed by crowding stress and decapitated. The panels above show representative immunoblots showing the expression of nNOS (**a**) and iNOS (**b**) in the hypothalamus. Graphs represent the means ± SEM of 10–12 rats per group. Data were assessed by one-way ANOVA followed by Newman’s test: ^++^*p* < 0.01 and ^+++^*p* < 0.001 vs. non-stressed control group; * *p* < 0.05 vs. isolation stress

In the HYPO, IS significantly increased the expression of nNOS protein compared with control level (*F*_(4, 32)_ = 7.26, ^++^*p* < 0. 01) (Fig. 7a) and subsequent CS for 3 days mitigated this increase. CS for 7 days did not alter the IS-induced nNOS protein level; however, CS for 14 days significantly enhanced the IS-induced nNOS level (*F*_(3,51)_ = 4.972, *p* = 0.0044, *p < 0.05) (Fig. 7a).

In the HYPO, IS slightly increased iNOS protein level compared with control level but was significantly increased following subsequent CS for 3 days (*F*_(3,36)_ = 11.12, *p < 0.05). A more extended period of CS (7 days) caused a significant decline in the IS-induced iNOS protein level *p < 0.05 (Fig. 7b).

**Fig. 8:**
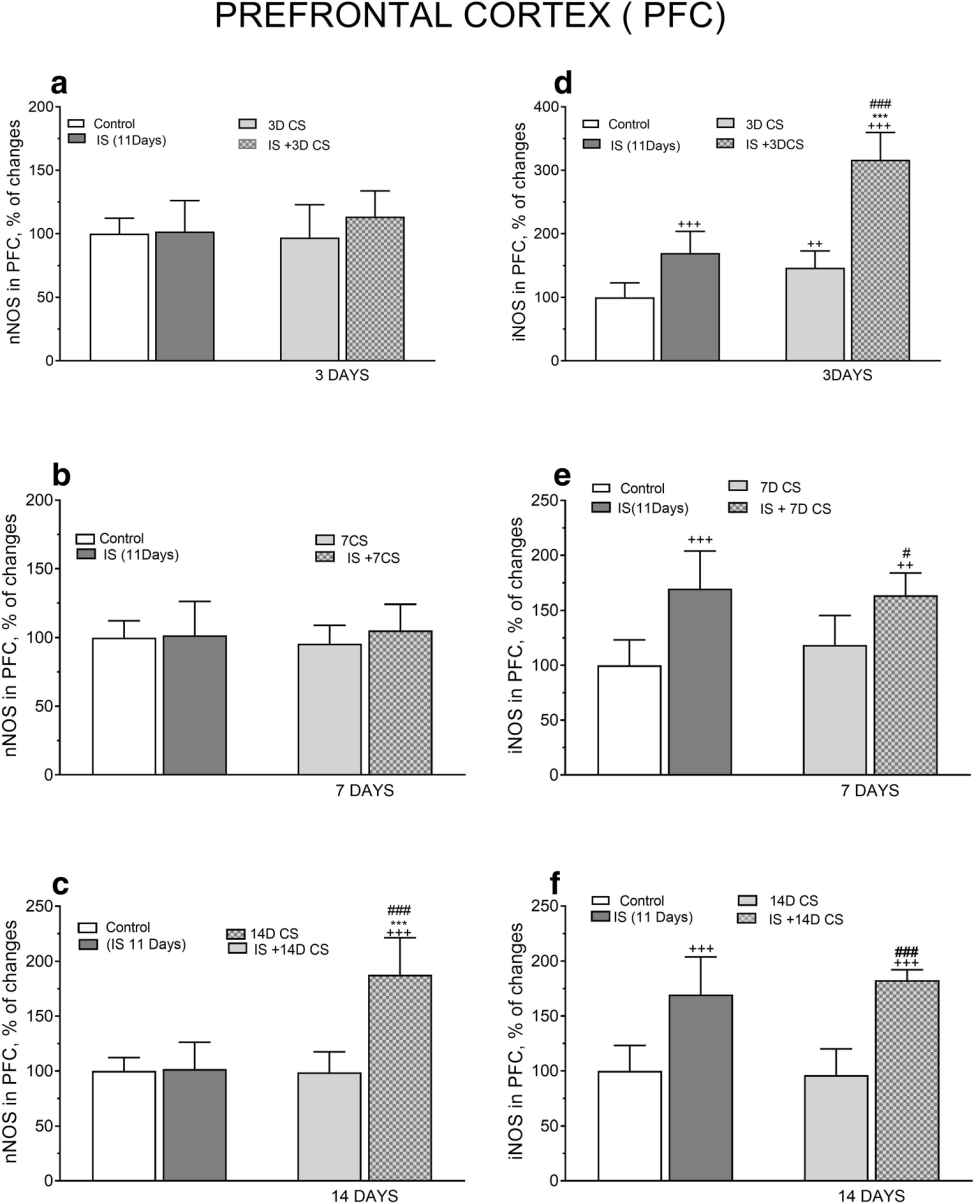
Comparison of the effect of isolation stress (IS) (for 11 days), crowding stress (CS) for 3 (**a**, **d**), 7 (**b**, **e**), and 14 (**c**, **f**) days, and IS + CS (for 3, 7, and 14 days) on nNOS (**a**, **b**, **c**) and iNOS (**d**, **e**, **f**) levels in the prefrontal cortex. Graphs represent the means ± SEM of 10–12 rats per group. Values are expressed as the mean ± SEM, *n* = 10–12 and were analyzed by two-way ANOVA and post hoc Tukey’s multiple comparison test: ^++^*p* < 0.01 and ^+++^*p* < 0.001 vs. non stressed control group; ***p < 0.001vs. IS; ^#^*p* < 0.05 and ^###^*p* < 0.001 vs. CS

In the PFC, IS alone for 11 days did not alter the expression of nNOS protein. Similarly, prior IS followed by 3 days of CS did not change the nNOS protein level. In the PFC, two-way ANOVA did not reveal significant interaction effects between IS (11 days) and subsequent CS for 3 days in nNOS protein expression (*F*_(1,43)_ = 1.093, *p* = 0.3017) (Fig. 8a). Similarly, there was no significant interaction between IS and subsequent CS for 7 days in the expression of nNOS protein (*F*_(1,38)_ = 0.3837, *p* = 0.5393) (Fig. 8b). However, there was a significant interaction between IS and subsequent CS for 14 days in the expression of nNOS protein (*F*_(1,41)_ = 33.05, *p* < 0.0001), effect of IS (*F*_(1,41)_ = 35.7, *p* < 0.0001), effect of CS (*F*_(1,41)_ = 31.12, *p* < 0.0001). Post hoc Tukey’s multiple comparison test revealed that both IS for 11 days and subsequent CS for 14 days participated equally and significantly (***p < 0.001vs. IS and ^###^*p* < 0.001 vs.14D CS; in this interaction) (Fig. 8c).

In the PFC, a two-way ANOVA revealed significant interaction between IS and successive CS for 3 days resulting in a very robust increase in the expression of iNOS protein (*F*_(1,42)_ = 24.15, *p* < 0.0001), with a significant increase in both IS (*F*_(1,42)_ = 137.7, *p* < 0.0001) and CS (*F*_(1,42)_ = 90.47, *p* < 0.0001) components. Post hoc Tukey’s multiple comparison test demonstrated significant and equal participation of both IS (***p < 0.001 vs. IS and CS ^###^*p* < 0.001 vs. 3D CS in this interaction) (Fig. 8d).

Two-way ANOVA revealed there was no interaction between IS and subsequent CS for 7 days in the expression of iNOS protein (*F*_(1,43)_ = 2.066, *p* = 0.1578), but significant increase of iNOS IS (*F*_(1,43)_ = 44.81, *p* < 0.0001), and successive CS for 7 days (*F*_(1,43)_ = 0.5328, *p* = 0.4694). Post hoc Tukey’s multiple comparison test showed strong participation of IS in inducing a final significant increase in iNOS protein expression ^#^*p* < 0.05 vs. 7 days CS (Fig. 8e).

Two-way ANOVA did not reveal any interaction between IS and subsequent CS for 14 days in the expression of iNOS protein (*F*_(1,40)_ = 0.9682, *p =* 0.3311). However, the final effect of IS was significant (*F*_(1,40)_ = 82.38, *p* < 0.0001), but not the effect of CS (*F*_(1,40)_ = 0.2855, *p* = 0.5961). Tukey’s multiple comparison test demonstrated significant participation of IS in the increased expression of iNOS protein ^###^*p* < 0.001 vs. 14D CS (Fig. 8f).

**Fig. 9:**
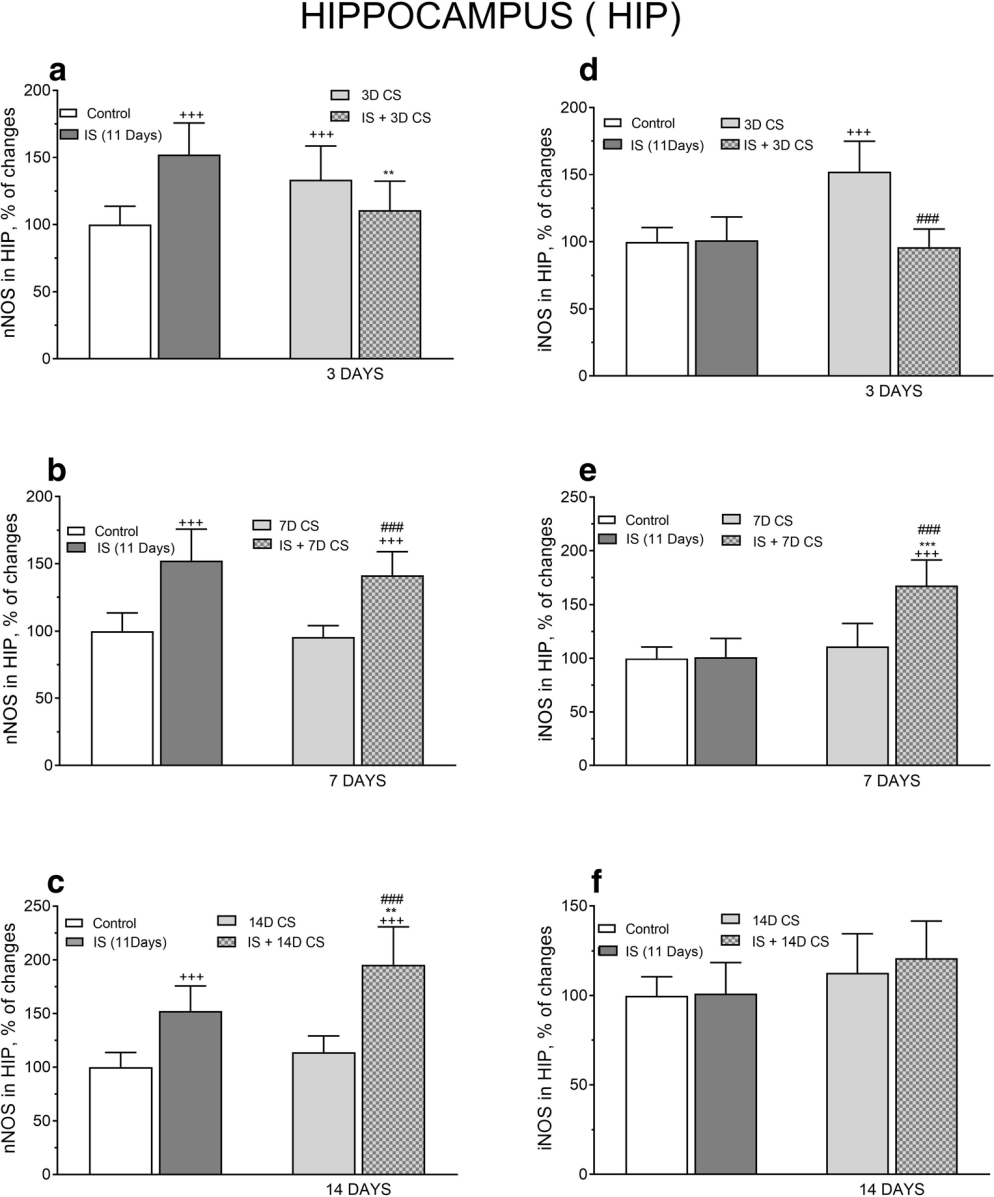
Comparison of the effect of isolation stress (IS) (for 11 days), crowding stress (CS) for 3 (**a**, **d**), 7 (**b**, **e**), and 14 days (**c**, **f**), and IS + CS (for 3, 7, and 14 days) on nNOS (**a**, **b**, **c**) and iNOS (**d**, **e**, **f**) levels in the hippocampus. Graphs represent the means ± SEM of 10–12 rats per group. Values are expressed as the mean ± SEM, *n* = 10–12, and were analyzed by two-way ANOVA and post hoc Tukey’s multiple comparison test: ^+++^*p* < 0.001 vs. non stressed control group; **p < 0.01 and *** *p* < 0.001 vs. IS; ^###^*p* < 0.001 vs. CS

In the HIP, two-way ANOVA revealed a significant interaction between IS and subsequent CS for 3 days in the expression of nNOS protein (*F*_(1,40)_ = 31.41, *p* < 0.0001) including a significant effect of IS (*F*_(1,40)_ = 4.893, *p* = 0.0327), but not the effect of CS (*F*_(1,40)_ = 0.395, *p* = 0.5333). Post hoc Tukey’s multiple comparison test showed that this interaction resulted from a significant increase of nNOS protein expression induced by IS (**p < 0.01 vs. IS) (Fig. 9a). There was still no interaction between IS and subsequent CS (7 days) in the expression of nNOS protein (*F*_(1,37)_ = 0.3579, *p* = 0.5533), although prior IS significantly increased nNOS protein expression (*F*_(1,37)_ = 80.72, *p* < 0.0001; ^###^*p* < 0.001 vs 7D CS) (Fig. 9b). However, there was marked interaction between IS and CS (14 days) resulting in an increased expression of nNOS protein (*F*_(1,42)_ = 4.737, *p* < 0.0352), effect of IS (*F*_(1,42)_ = 99.27, *p* < 0.0001), and effect of CS (*F*_(1,48)_ = 17.9, *p* = 0.0001). Tukey’s multiple comparison test indicated both IS and CS participated in this interaction (**p < 0.01vs. IS; ^###^*p* < 0.001 vs. 14D CS) (Fig. 9c), although the effect of IS was more robust.

In the HIP, two-way ANOVA revealed a significant interaction between IS and subsequent CS for 3 days in the expression of iNOS protein (*F*(_1,42_) = 28.19, *p* = 0.0001) and significant effect of IS (*F*_(1,42)_ = 25.82, *p* = 0.0001) and effect of CS (*F*_(1,42)_ = 19.04, *p* = 0.0001). Tukey’s multiple comparison test indicated that the interaction resulted from a significant decrease in iNOS protein level by CS (^###^*p* < 0.001 vs. 3D CS) (Fig. 9d).

Two-way ANOVA revealed a significant interaction between IS and subsequent CS for 7 days resulting in a robust increase in iNOS protein expression (*F*_(1,55)_ = 23.72, *p* < 0.0001). Also, significant positive effects on iNOS protein levels were observed after IS (*F*_(1,55)_ = 25.89, *p* < 0.0001) and CS (*F*_(1,55)_ = 46.98, *p* < 0.0001). Post hoc Tukey’s multiple comparison test demonstrated highly significant participation of IS and CS in the increased expression of iNOS protein (***p < 0.001 vs IS, ^###^*p* < 0.01 vs. 7D CS) (Fig. 9e).

Exposure to CS for 14 days post IS revealed no interaction between IS and CS in iNOS protein expression (*F*_(1,48)_ = 0.3572, *p =* 0.5529) and effect of IS (*F*_(1,48)_ = 0.6529, *p* = 0.4230) and CS (*F*_(1,48)_ = 7.768, *p* = 0.0076) (Fig. 9f).

**Fig. 10:**
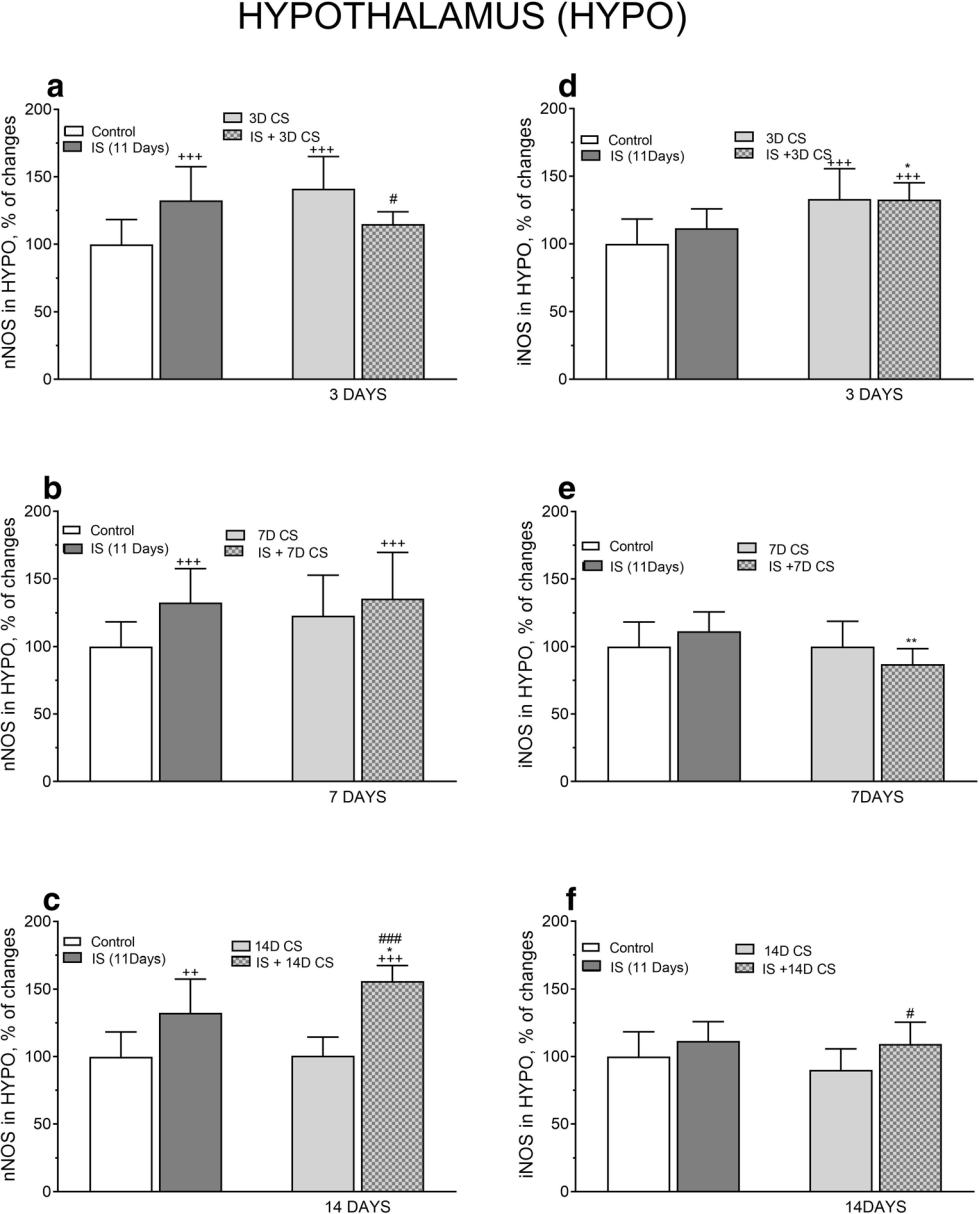
Comparison of the effect of isolation stress (IS) (for 11 days), crowding stress (CS) for 3 (**a**, **d**), 7 (**b**, **e**), and 14 days (**c**, **f**), and IS + CS (for 3, 7, and 14 days) on nNOS (**a**, **b**, **c**) and iNOS (**d**, **e**, **f**) levels in the hypothalamus. Graphs represent the means ± SEM of 10–12 rats per group. Values are expressed as the mean ± SEM, *n* = 10–12, and were analyzed by two-way ANOVA and post hoc Tukey’s multiple comparison test: ^++^*p* < 0.01 and ^+++^*p* < 0.001 vs. non stressed control group; *p < 0.05 and **p < 0.01 vs. IS; ^#^*p* < 0.05 and ^###^*p* < 0.01 vs. CS

In the HYPO, two-way ANOVA indicated a significant interaction between IS and subsequent 3 days of CS in the expression of nNOS protein (*F*_(1,49)_ = 22.3, *p* < 0.0001). The effects of IS (*F*_(1,49)_ = 0.2618, *p* = 0.6112) and CS (*F*_(1,49)_ = 3.63, *p* = 0.0626) were not significant. Tukey’s multiple comparison test showed a significant reduction in nNOS protein expression (^#^*p* < 0.05 vs. 3D CS) (Fig. 10a).

Two-way ANOVA did not reveal any interaction between IS (11 days) and subsequent CS for 7 days in the expression of nNOS protein (*F*_(1,47)_ = 1.554, *p* = 0.2188), both the effects of IS (*F*_(1,47)_ = 7.789, *p* = 0.0076), and those of CS (*F*_(1,43)_ = 2.561, *p* = 0.1162) on nNOS protein level were not significant (Fig. 10b). Two-way ANOVA revealed a marked interaction between IS and subsequent CS for 14 days in the expression of nNOS protein (*F*_(1,57)_ = 4.561, *p* = 0.0370). Post hoc Tukey’s test showed a significant increase of nNOS protein expression after IS (*p < 0.05vs. IS and ^###^*p* < 0.001 vs. 14D CS). Both effects of IS (*F*_(1,57)_ = 67.42, *p* < 0.0001) and CS (*F*_(1,57)_ = 5.19, *p* = 0.0265) on the expression of nNOS in the HYPO were significant (Fig. 10c).

Two-way ANOVA did not reveal any interaction between IS and subsequent CS for 3 days in the expression of iNOS protein (*F*_(1,50)_ = 1.56, *p* = 0.2175). Tukey’s multiple comparison test demonstrated a significant increase in iNOS protein level after IS and subsequent CS for 3 days (*p < 0.05 vs. IS), and the effect of CS (*F*_(1,50)_ = 32.26, *p* < 0.0001) was significant (Fig. 10d).

In the HYPO, IS significantly interacted with successive CS for 7 days in the expression of iNOS protein (*F*_(1,49)_ = 7331, *p* = 0, 0093). Multiple comparison test demonstrated a significant decrease of iNOS protein level after IS and 7 days of CS (**p < 0.01 vs. IS) (Fig. 10e).

However, CS for 14 days after prior IS revealed no interaction in the expression of iNOS protein (*F*_(1,58)_ = 0.784, *p =* 0.3796), although IS significantly increased CS-induced iNOS level (*F*_(1,58)_ = 12.44, *p* = 0.0008) (Fig. 10f).

### Effect of Chronic IS on Plasma IL-1β, ACTH, and CORT Levels

**Fig. 11:**
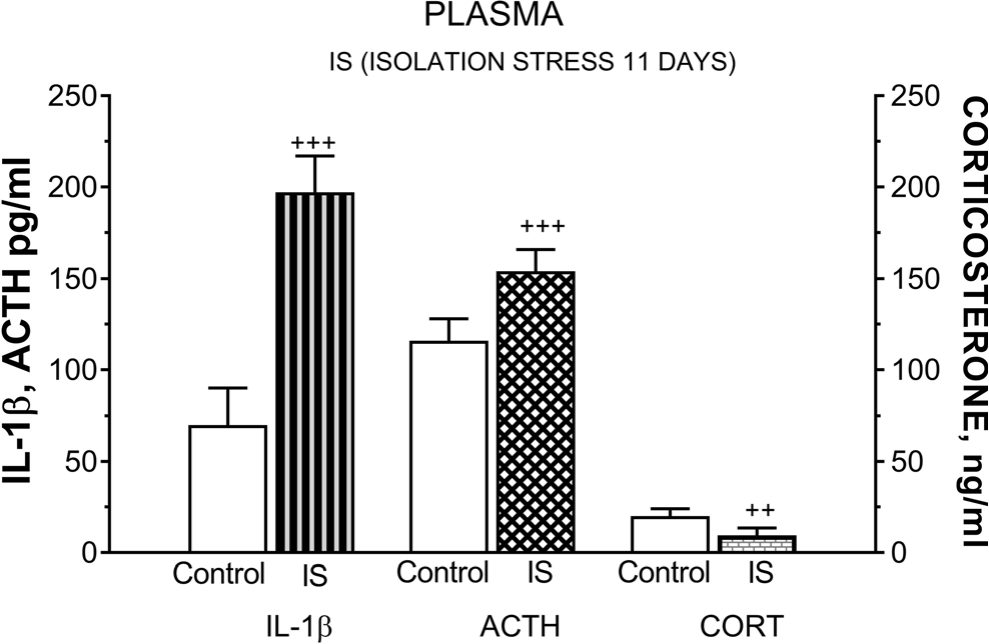
The effect of isolation stress (IS) for 11 days on plasma ACTH, corticosterone (CORT), and interleukin-1β (IL-1β) levels. Rats exposed to isolation stress were kept 1 per cage for 11 consecutive days and decapitated. Graphs represent the mean ± SEM, *n* = 10–12 rats per group. Data were analyzed by Student’s *t* test: ^++^*p* < 0.01 and ^+++^*p* < 0.001 vs. non-stressed control group

Exposure of rats to IS for 11 days significantly increased both plasma IL-1β (*t* = 3.272, df = 12, *p* < 0.001) and ACTH (*t* = 5.216, df = 17, ^+++^*p* < 0.001) levels compared with controls. By contrast, rats exposed to SI exhibited a significant decrease in plasma CORT level compared with non-stressed rats (*t* = 2.336, df = 15, ^++^*p* < 0. 01) (Fig. 11).

### Effect of Chronic Social IS on CS-Induced Plasma IL-1β, ACTH, and CORT Levels

**Fig. 12:**
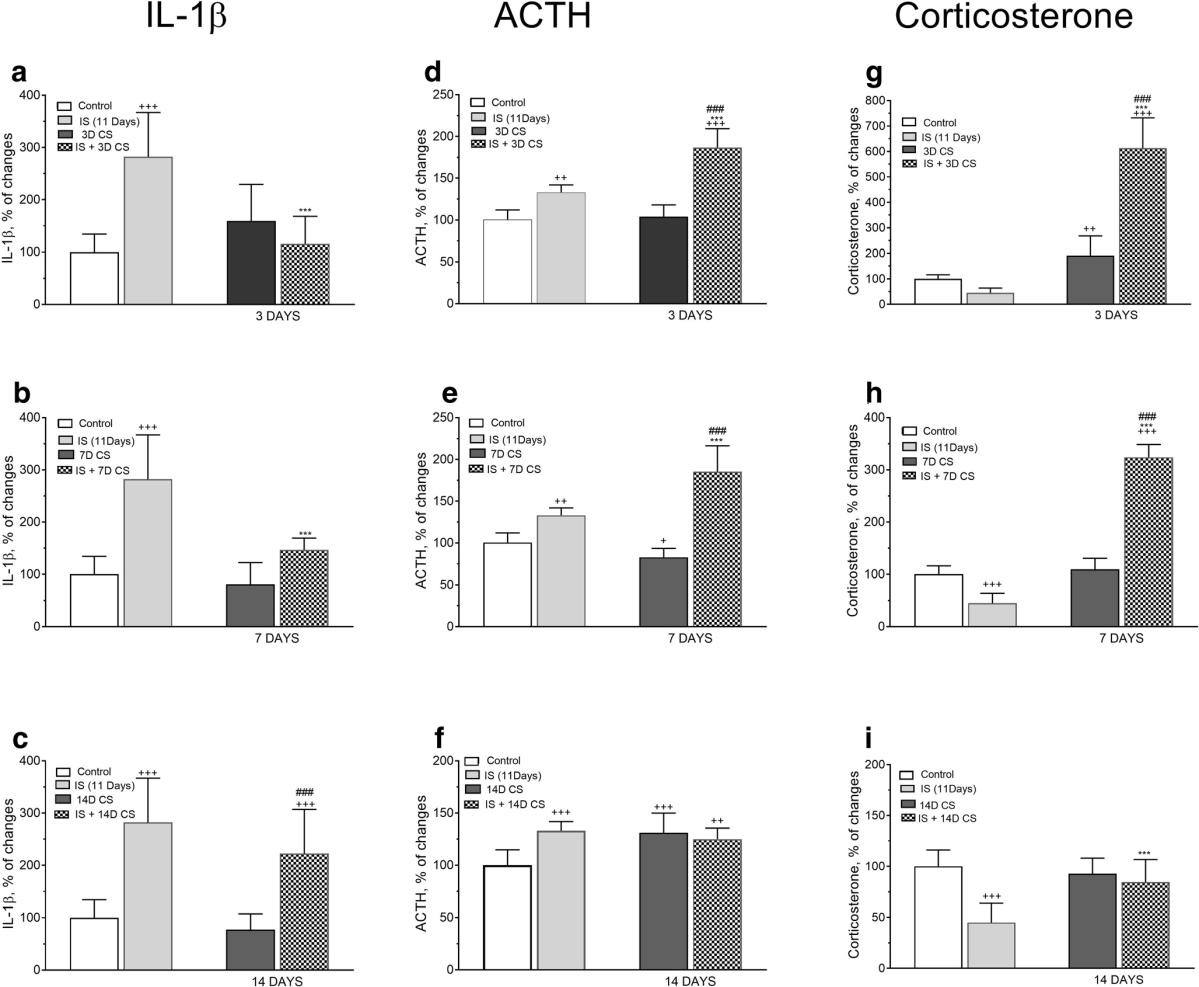
Comparison of the effect of isolation stress (IS) (for 11 days), crowding stress (CS) for 3 (**a**, **d**, **g**), 7 (**b**, **e**, **h**), and 14 days (**c**, **f**, **i**), and IS + CS (for 3, 7, and 14 days) on IL-1β (**a**, **b**, **c**), ACTH (**d**, **e**, **f**), and corticosterone levels (**g**, **h**, **i**) in plasma. Graphs represent the means ± SEM of 10–12 rats per group. Values are expressed as the mean ± SEM, *n* = 10–12 and were analyzed by two-way ANOVA and post hoc Tukey’s multiple comparison test: ^+^*p* < 0.05, ^++^*p* < 0.01, ^+++^*p* < 0.001 vs. non stressed control group; ***p < 0.001 vs. IS; ^###^*p* < 0.001 vs. CS

Two-way ANOVA revealed a highly significant interaction between isolation stress for 11 days and successive CS for 3 days resulting in decreased plasma IL-1β protein level 3D CS (*F*_(1,40)_ = 36.92, *p* < 0.0001). IS significantly lowered plasma IL-1β level induced by CS (*F*_(1,40)_ = 13.81, *p* = 0.0006) and effect of CS (*F*_(1,40)_ = 8.313, *p* = 0.0063). Post hoc Tukey’s test showed a significant decrease in the expression of IL-1β protein level after IS and subsequent CS for 3 days (***p < 0.001 vs. IS, ^+++^*p* < 0.001 vs. control) (Fig. 12a).

A longer period of CS (7 days) revealed significant interaction (*F*_(1,31)_ = 11.41, *p* = 0.0019), IS (*F*_(1,31)_ = 51.81, *p* < 0.0001) and CS (*F*_(1,31)_ = 20.11, *p* < 0.0001). Post hoc Tukey’s test showed a significant decrease in the expression of IL-1β protein level after IS and subsequent CS for 7 days (***p < 0.001 vs. IS and ^+++^*p* < 0.001 vs. control) (Fig. 12b). However, extended periods of CS (14 days) following IS did not reveal any interaction in the expression of IL-1β protein level (*F*_(1,38)_ = 0.8792, *p* = 0.3543), IS (*F*_(1,38)_ = 69.15, *p* < 0.0001), and CS (*F*_(1,38)_ = 4.376, *p* = 0.0432). Post hoc Tukey’s test showed a significant increase in the expression of IL-1β protein level after IS and subsequent CS for 7 days (^###^*p* < 0.001 vs. CS ^+++^*p* < 0.001 vs. control) (Fig. 12c).

Plasma ACTH and CORT were significantly altered by chronic psychosocial stressors of social isolation and social crowding. Two-way ANOVA showed highly significant interaction between IS and successive CS for 3 days (*F*_(1,31)_ = 23.94, *p* < 0.0001), with a considerable increase of IS (*F*_(1,31)_ = 126.2, *p* < 0.0001) and CS component (*F*_(1,31)_ = 30.96, *p* = 0.0001). Post hoc Tukey’s multiple comparison test revealed ^+++^*p* < 0.001 vs. control, ***p < 0.01 vs. IS, and ^###^*p* < 0,001 vs. 3D CS (Fig .12d).

Likewise, a longer CS for 7 days after IS showed significant interaction resulting in increased plasma ACTH level (*F*_(1,31)_ = 32.6, *p* < 0.0001) with significant effect of IS (*F*_(1,31)_ = 121.2, *p* < 0.0001) and CS (*F*_(1,31)_ = 7.995, *p* = 0.0081). Post hoc Tukey’s multiple comparison test revealed **p < 0.01vs. IS and ^###^*p* < 0.001 vs. 7D CS (Fig. 12e).

However, longer successive CS for 14 days after IS revealed significant interaction in increasing plasma ACTH level (*F*_(1,39)_ = 18.36, *p* = 0.0001) due to increased IS component (*F*_(1,39)_ = 8.615, *p* = 0.0056) and effect of CS (*F*_(1,39)_ = 6.387, *p* = 0.0157) (Fig. 12f).

Two-way ANOVA showed a significant interaction between IS and successive CS for 3 days in inducing a robust increase in plasma CORT level (*F*_(1,39)_ = 110.7, *p* < 0.0001) with significant effect of IS (*F*_(1,39)_ = 65.48, *p* < 0.0001) and CS (*F*_(1,39)_ = 212.3, *p* < 0.0001). Post hoc Tukey’s multiple comparison test revealed ***p < 0.001 vs. IS and ^###^*p* < 0,001 vs. 3D CS (Fig. 12g).

A similar positive interaction effect on plasma CORT level was observed after a longer successive CS (7 days) following prior IS, interaction effect IS/7D CS + 7D CS (*F*_(1,32)_ = 392.2, *p* < 0.0001), effect of IS (*F*_(1,32)_ = 137.5, *p* < 0.0001), and effect of CS (*F*_(1,32)_ = 449.3, *p* < 0.0001). Post hoc Tukey’s multiple comparison test revealed ^+++^*p* < 0.001 vs. control, ***p < 0.001 vs. IS, and ^###^*p* < 0.001 vs. 7D CS (Fig. 12h). Two-way ANOVA also showed a significant but lesser interaction after longer CS periods (14 days) following IS. Interaction effect 14D CS/IS + 14D CS (*F*_(1,35)_ = 15.1, *p =* 0.0004), with effect of IS (*F*_(1,35)_ = 27.59, *p* = 0.0001) and effect of CS (*F*_(1,35)_ = 7.221, *p* = 0.011); Post hoc Tukey’s multiple comparison test revealed ***p < 0.001 vs. IS (Fig. 12i).

### Isolation Stress-Induced Changes in nNOS and iNOS Protein Levels in Brain Structures and Plasma IL-1β, ACTH, and CORT Levels Induced by Followed CS

Table 1 presents data from two-way ANOVA and the effect of chronic IS (11 days) followed by CS-induced changes in nitric oxide synthases in PFC, HIP, and HYPO and in plasma IL-1β, ACTH, and CORT levels.

**Table 1:**
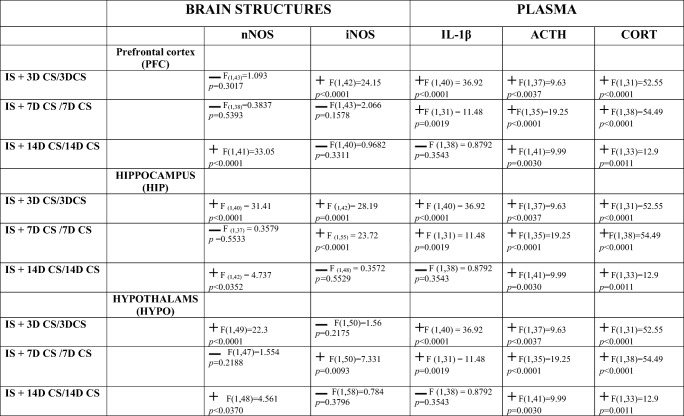
Changes in nNOS and iNOS protein levels in brain structures and changes in IL-1β, ACTH, and corticosterone (CORT) plasma levels in animals subjected to isolation stress (IS) for 11 days followed by crowding stress (CS) for 3, 7, and 14 days in comparison with the animals that were exposed to crowding stress for 3, 7, and 14 days alone. Two-way ANOVA showed **+** interaction  no interaction

In summary, our results indicate that chronic IS significantly interact in the increase of plasma ACTH and CORT levels after CS for 3, 7, and 14 days. The similar increase appeared in plasma IL-1β levels after 3 and 7 days of CS. In addition, prior IS significantly interacts with iNOS protein expression in the PFC and HIP after CS for 3 days and in the HYPO after 7 days of CS. IS also significantly interacts in increasing nNOS protein level after CS for 14 days in the PFC, HIP, and HYPO after CS for 3 days. The HIP appeared the most sensitive brain structure in the interaction of IS on NOS and plasma IL-1β, ACTH, and CORT levels with subsequent CS for 3, 7, and 14 days. The mechanism of IS-induced changes in brain structures and plasma after successive CS requires further study.

## Discussion

Previous studies from our laboratory showed that in the PFC, the most stress-sensitive brain structure, mild social CS did not alter the expression of the nNOS isoenzyme in 3–14 days (Gądek-Michalska et al. 2017). Similarly, the immunolocalization of nNOS indicated a robust increase in NO levels in the HIP and striatum relative to the cortex. The expression of nNOS in the cerebral cortex in vivo was transient and very low, compared with the HIP (Lourenço et al. 2014). The constitutively expressed nNOS is the major isoenzyme in the HIP responsible for NO production; it may act as a modulator directly or by acting on other neural networks in the brain through cyclic guanosine monophosphate (cGMP)-dependent mechanism (Guix et al. 2005; McLead et al. 2001; Garthwaite 2008). In our study, the social CS-induced responses of NOS may be mediated by neuronal nitrergic and oxidative/nitrosative pathways in the PFC and HIP (Busnardo et al. 2019; Zlatković and Filipović 2013; Filipović et al. 2017).

We observed that CS for 3 days significantly enhanced iNOS protein expression in the PFC, HIP, and HYPO, more than nNOS, but did not alter iNOS protein level after a longer duration of CS (7 and 14 days). Chronic social CS has been shown to increase NO expression in NOS-positive neurons located in the HIP, cerebral cortex, and glial cells (Vincent et al. 1998). Also, astrocytes surrounding synaptic elements can release NO and modulate synaptic transmission (Amitai 2010). Repeated restraint stimulated the NOS/NO pathway in the HYPO by selective and long-lasting enhancement of excitatory synaptic inputs into CRH synthesizing parvocellular neuroendocrine cells partially by a NOS-dependent mechanism (Kusek et al. 2017). Our present results indicate that in the PFC 3 days or longer periods of psychosocial CS did not alter nNOS but significantly increased iNOS protein level after 3 days of CS. The PFC, HIP, and HYPO participated in the modulation of CS responses, after 3 days during which time there was a significant increase in iNOS protein expression in these brain regions. Neither nNOS nor iNOS protein levels were markedly altered after longer durations of CS, although a single stress episode delayed modifications of the NO pathway in the hippocampal formation (Echeverry et al. 2004). NO generated by nNOS during chronic mild stress suppresses hippocampal neurogenesis and inhibits signaling in the brain (Zhou et al. 2007). Chronic psychosocial IS for 11 days itself significantly increased the expression of the nNOS protein and probably NO synthesis in the HIP and HYPO and may mediate the functional adaptation of the nNOS/NO pathway in these structures.

By contrast, we observed that iNOS protein expression significantly increased in the PFC but not in the HIP or HYPO, suggesting that the iNOS/NO pathway is almost selectively activated in the PFC during central adaptation to chronic stimulation during severe psychosocial IS. Our results indicate that prior social IS coupled with subsequent CS affects nNOS and iNOS protein expression differently and is dependent on the brain structure, duration of CS exposure, and intensity of the stressor.

A two-way ANOVA was used to determine the strength of interaction if any after prior chronic IS (11 days) followed by subsequent CS for 3, 7, and 14 days in the modulation of nNOS and iNOS protein expression in the PFC, HIP, and HYPO. In the PFC, there was no apparent interaction in the level of nNOS after 3 and 7 days of CS following prior IS; however, there was significant interaction of IS/IS + CS after a longer duration of CS (14 days). Similarly, a robust interaction was seen in iNOS protein expression after 3 days of CS. This interaction of combined stress in enhancing the expression of nNOS and iNOS protein resulted from the stimulatory action of IS and CS.

In the HIP, the combined stress of IS and subsequent CS for 3 and 7 days significantly interacted in the expression of iNOS protein by reducing iNOS protein level after 3 days of CS and strong enhancement after 7 days. Previous and recent studies suggest that chronic IS in rats and mice mitigate the negative impact of NO in the brain and HPA axis functioning following subsequent acute stressors (Zlatković and Filipović 2013; Haj-Mirzaian et al. 2016a).

In the HYPO, an interaction was evident in the expression of nNOS protein 3 days after CS following initial IS as evidenced by the significant decrease of CS level and after a longer CS duration 14 days, by increased IS and CS action.

Our results indicate that prior IS enhances in general, iNOS level induced by following psychosocial CS for 3, 7, and 14 days in the PFC and HYPO but reverses these responses in the HIP, the highly sensitive brain region in psychosocial responses.

Dysregulation of HPA axis activity after chronic stress exposure may result from IL-1β and NO overproductions.

Chronic IS-induced overexpression of iNOS and NO overproduction are attenuated by lithium (Haj-Mirzaian et al. 2016b).

Chronic social stressors are known to upregulate HPA axis by increasing CRH cell number and CRH mRNA expression in the hypothalamic PVN (Backström and Winberg 2013). The distribution of CRH across many brain areas indicates that the CRH system also functions outside the classical HPA axis (Hostetler and Ryabinin 2013). CRH has also been shown to have a role in social memory. The PFC modulates conflicting inputs of working memory and information from the social environment and coordinates complex neuro-hormonal and behavioral responses in humans and animals (Hostinar et al. 2014). In this respect, the central CRH system with its high density of receptors in the brain (Barra de la Tremblaye and Plamondon 2016) may have a role in perception of signals from the limbic system (Herman et al. 2003, 2005) during chronic social isolation and social CS to discriminate and coordinate proper defensive responses. We found that IS alone for 11 days considerably enhanced plasma IL-1β protein expression (threefold) and significantly increased ACTH level but declined CORT level. Chronic social isolation of rats for 21 days alone or in combination with acute stress also reduced serum CORT levels (Zlatković et al. 2014). This decrease in the level of CORT level could suggest exhaustion of the adrenal cortex after chronic IS. A similar blunted cortisol response to a psychosocial stressor was observed during major depressive disorder in humans (Simeon et al. 2007). Chronic social isolation induces adrenal hypertrophy resulting in elevated ACTH and CORT levels (Krügel et al. 2014; Perelló et al. 2006). Chronic IS, like chronic variable stress (CVS), increased the synaptic excitability of PVN CRH neurons of the pituitary (Serra et al. 2005, 2007; Franco et al. 2016) and markedly increased the functional response of the HPA axis to an acute stressful stimulus (foot shock) (Serra et al. 2007).

Our results indicate that chronic IS significantly increased plasma IL-1β level after 7 and 14 days of subsequent CS, while ACTH and CORT levels increased before IL-1β after 3 and 7 days of subsequent CS. Chronic IS enhanced the responsiveness of the brain structures and plasma HPA axis to new stimuli more in isolated than in group-housed rats. The enhanced effects of acute stress may be related to the sensitization of the HPA axis that develops as an adaptive response to chronic stress.

During major stressful activation of the HPA axis, the increased ACTH signals may considerably enhance adrenal glucocorticoid responsiveness (Russell et al. 2015). Stress-induced CORT response may affect emotional processing and modulate differential activation of brain regions, particularly HIP, PFC, and HYPO. Although no significant correlation was observed between plasma NO and CRH levels in depression, both levels were increased (Lu et al. 2018).

## Conclusions

Our data indicate that social CS is a weak stimulator of nNOS expressing systems in the PFC, whereas in the HIP and HYPO CS significantly enhances the expression of nNOS and iNOS proteins after 3 days of CS. Both nNOS and iNOS isoenzymes in the PFC, HIP, and HYPO participate in chronic IS- and CS-induced generation of NO in a structure and time-dependent manner. Social isolation stress strongly affects the subsequent social CS-induced effects of nNOS in brain structures and plasma HPA axis responses. For many of the common experimental animals are highly social species (rats, mice, primates), group housing is more naturalistic but social isolation is a potent stressor, with long-term consequences on the stress-related brain structures and HPA axis. Our present and former studies show that prior social IS coupled with subsequent CS affects nNOS and iNOS protein expression differently depending on the brain structure, duration of CS exposure, and intensity of the stressor.

## Abbreviations

ACTH: Adrenocorticotropic hormone
cGMP: Cyclic guanosine monophosphate
CNS: Central nervous system
CORT: Corticosterone
CRH: Corticotropin-releasing hormone
HPA: Hypothalamic-pituitary-adrenal
IL-1β: Interleukin-1β
iNOS: Inducible nitric oxide synthase
mRNA: Messenger ribonucleic acid
nNOS: Neuronal nitric oxide synthase
NO: Nitric oxide
PFC: Prefrontal cortex
PVN: Paraventricular nuclei
CS: Crowding stress
IS: Isolation stress
HIP: Hippocampus
HYPO: Hypothalamus
GCs: Glucocorticoids
CVS: Chronic variable stress
CMS: Chronic mild stress
